# Microenvironment and the progress of immunotherapy in clinical practice of NSCLC brain metastasis

**DOI:** 10.3389/fonc.2022.1006284

**Published:** 2023-01-24

**Authors:** Mengqing Xie, Chunxia Su

**Affiliations:** Department of Oncology, Shanghai Pulmonary Hospital and Thoracic Cancer Institute, School of Medicine, Tongji University, Shanghai, China

**Keywords:** NSCLC, brain metastasis, immunotherapy, organ-specific, time

## Abstract

One of the most frequent distant metastases of lung cancer occurs in the brain. The average natural survival duration for patients with lung cancer who have brain metastases is about 1 to 2 months. Knowledge about brain metastases is currently restricted since they are more difficult to acquire than other metastases. This review begins with an analysis of the immune microenvironment of brain metastases; focuses primarily on the functions of microglia, astrocytes, neurons, and tumor-infiltrating lymphocytes in the microenvironment of brain metastases; and offers an atlas of the immune microenvironment of brain metastases involving significant cells. In an effort to give researchers new research ideas, the study also briefly covers how immunotherapy for non-small cell lung cancer with brain metastases is currently faring.

## 1 Introduction

Lung cancer is the leading cause of cancer-related deaths worldwide. The majority of lung cancers (85%) are non-small cell lung cancers (NSCLCs), which are typically found at an advanced stage and have a dismal 5-year overall survival (OS) of 15%–21% (1). The greatest worry in advanced NSCLC is metastasis, which is typically defined as a process in which malignant cells migrate from the initial location to distant sites and indicates considerable mortality and worse quality of life (2). The metastasis of NSCLC has complex organ-tropism mechanisms, mainly involving the organs of the brain, liver, and bone (3). Due to the challenges in obtaining the tissue, the field of brain metastasis (BM) is by far the least explored of these sites. BM affects 15%–43% of lung cancer patients, with an OS of about 1–2 months for untreated individuals (4, 5). Systemic therapy such as chemotherapy and topical medications such as surgical resection, whole-brain radiation therapy, and stereotactic radiosurgery (SRS) are routinely performed, with limited improvement of OS for patients (6). Immunotherapy provides insight into this dilemma.

The landscape of advanced NSCLC has changed dramatically as a result of the findings from clinical trials like KEYNOTE-024 (7) and KEYNOTE-189 (8). However, the fact that the efficacy of therapeutic agents against BM was discouraging excluded the enrollment of patients with BM in immune checkpoint inhibitor (ICI) clinical trials, resulting in the blank of NSCLC with BM in the ICI field. The impermeability of the blood–brain barrier (BBB) and the complicated location of brain metastases restrict the use of treatment medicines and put a cloud over BM patients (9). The BBB, a structure peculiar to the central nervous system (CNS) and made up of astrocytes, pericytes, and endothelial cells with tight connections, protects the CNS from circulating toxins and inflammation. The BBB has historically been assumed to be difficult for therapeutic medicines with large molecular sizes and limited solubility to pass, making them impossible to exhibit the anti-tumor effect of these regimens in the CNS (10). However, more ICIs have recently shown promise in the routine care of BM patients, pointing to the possibility of a remote interaction between tumor cells and the distinct tumor immune microenvironment (TIME) (11).

In this review, we will summarize the complex TIME in the brain parenchyma and the evolving scenario of the application of ICIs in BM patients to construct a comprehensive understanding of BM.

## 2 TIME and clinical practice

### 2.1 TIME

#### 2.1.1 Microglia/BMDMs is the main immune cells in the CNS

The CNS’s principal resident immune cell and the first line of innate immunity, the microglia, is an essential part of the CNS (12). Microglia, which are referred to as the “third element of the nervous system”, are only produced from progenitors found in the yolk sac and developed during the embryonic stage (13). Presenting throughout the CNS, mainly the gray matter (14), these myeloid lineage cells are estimated to constitute up to 5%–10% of the cellular composition (15–17).

The CNS’s homeostasis and the growth of the brain are primarily supported by microglia. Evidence from time-lapse recording suggested that microglia might scan the brain parenchyma during neuronal activation and play a role in synapse modulation (18, 19). In addition, microglia participate in a variety of physiological activities, including the removal of cellular waste and neurogenesis (20, 21). Microglia typically undergo a transformation toward an active phenotype in response to pathogens and cancerous cells as part of their defensive function, allowing them to monitor the microenvironment and keep an eye out for any potential disturbances (22). Pro- and anti-inflammatory cytokines and chemokines are usually released to modulate the scenario of infection or metastasis foci as well (23).

However, bone marrow-derived macrophages (BMDMs) are frequently recruited in the TIME of BM in addition to microglia (24). It is challenging to distinguish between microglia and BMDMs in part because there are few experimental methods and biomarkers available. The advancement of technology gives us opportunities to learn more in this area that have never existed before. Microglia is a differentiated cell that can self-renew (25), while BMDMs replenish through peripheral monocytosis (24). Under physiological settings, microglia appear in a ramified resting status, but when activated, they transform into hypertrophic and finally ameboid active forms (14). In contrast to microglia, BMDMs exhibit a roundish morphology while quiescent and turn stretched and elongated in M2 phenotypes (26), demonstrating the distinction. Microglia and BMDMs shared certain similar markers, such as CD11b, ionized calcium-binding adaptor molecule 1, CD68, and CX3C chemokine receptor 1, even though distinct ontogenesis gave the two different physical characteristics (27–30). The distinction between microglia and BMDMs is the subject of more widespread debate. Among the inflammatory of *T. gondii*, the analysis of mouse brain revealed that microglia were the CD11b^+^/CD45^low^ population, compared with the CD11b^+^/CD45^high^ for macrophage (31). More stable markers are urgently required due to the volatility of CD45 expression after the injury or disease. Transmembrane protein 119, which was not expressed in BMDMs, was discovered by Bennett et al. using a fluorescence-activated cell sorter in conjunction with RNA sequencing to identify microglia as a stable and reliable marker (32). Using Ccr2^RFP/+^ mice, in which monocytes infiltrating the brain are marked with a red fluorescent protein (RFP) and presenting with the result that RFP^+^ monocytes were negative for sialic acid-binding immunoglobulin-like lectin H (Siglec-H), Konishi et al. confirmed that Siglec-H was unique to microglia. Siglec-H, a transmembrane lectin, has also been discovered as a marker for microglia from developmental to mature stages by transcriptome analysis and immunohistochemistry (33, 34). All microglia expressed green fluorescent protein (GFP), while only approximately 5% of CNS-associated macrophages did, according to research by Buttgereit et al. using Sall1 reporter mice (Sall1^GFP/+^). This finding demonstrated that Sall1 was confined to microglia (35). Other markers have also demonstrated the capacity to differentiate between microglia and BMDMs such as CD49D/integrin subunit alpha 4, a marker exclusive to tumor-infiltrating BMDMs (24). However, it should be highlighted that knowledge of microglia and BMDMs is still in its infancy, necessitating a deeper examination of functional analysis in light of the complex situation.

Microglia/BMDMs are crucial to the CNS processes of metastasis, dormancy, and relapse. Clarifying the relationship between microglia/BMDMs and cancer cells is essential. BBB protects BM by acting as a guard to maintain homeostasis. Numerous microglia have been seen in the perivascular region, suggesting that they may be involved in the BBB’s regulation (36). By using the purinergic receptor P2Y, G-protein coupled, 12 (P2RY12) inhibitor clopidogrel, and P2RY12^−/−^ mice, Lou et al. confirmed that microglia could contribute to the resealing of BBB and keep its integrity in a P2RY12-dependent manner (37). Claudin-5 is initially expressed by microglia in order to make contact with endothelial cells and preserve the BBB’s integrity. Astrocytic end-feet are engulfed by activated microglia, which also promote persistent inflammation and BBB damage (38). Other pro-inflammatory cytokines including tumor necrosis factorα (TNF-α), interleukin-1β (IL-1β), and interleukin-6 (IL-6) are also used by microglia to maintain the integrity of the BBB (39).

As the most prevalent tissue-resident macrophage, microglia/BMDMs contribute to the preservation of the BBB as well as the destruction of cancer cells and the growth of BM. The polarization status of M1 microglia is characterized by secreting pro-inflammatory cytokines such as IL-1β, TNF-α, inducible nitric oxide synthase, and reactive oxygen species. M2-type microglia promote tumor growth and are activated by interleukin-4, interleukin-10, and transforming growth factor-β (40). The cytokines secreted by the different states of microglia/BMDMs played the opposite effect. This field needs to be further explored as well.

#### 2.1.2 Astrocyte play a bidirectional role in patients with BM

The largest and most prevalent cell type is the astrocyte, a star-shaped neuroglia that has five times as many cells as neurons. Recent research has demonstrated that the function of nearby neurons is matched by the astrocytes, which occur in intraregional heterogeneity (41, 42). Astrocytes perform a number of crucial tasks, such as ion homeostasis, neurotransmitter recovery, synapse formation, and BBB modulation, due to their diverse physiological activities and ubiquitous nature. Astrocytes cause morphological and transcriptomic alterations and turn into reactive astrocytes (RAs) in response to a stimuli, such as a disease or injury (43). RAs are traditionally characterized by high glial fibrillary acidic protein (44). Technology advancements have made it possible to define the status of RAs using cutting-edge methods like transcriptome analysis, and studies reveal that complex functional alterations occur in RAs across various disease models (45).

At the beginning of metastasis, astrocytes had the ability to cause Fas-dependent death in brain-tropic cells (46). However, it should be noted that RAs succumb to tumor progression because of the paracrine cytokine communication loops between them and cancer cells, which create a pro-tumorigenic environment. Seike et al. carried out co-culture studies based on the aforementioned theory, confirming the reciprocal connection between lung cancer cells and astrocytes. The researchers discovered that substances targeting tumor cells, such as macrophage migration inhibitory factor, interleukin-8, and plasminogen activator inhibitor-1, might activate RAs. Additionally, RAs may emit cytokines like IL-6, TNF-α, and IL-1β that may encourage the growth of cancer cells like PC-9, QG56, and EBC-1, which are generated from human lung cancer (47). The melanoma-to-brain metastasis evidenced the bidirectional signaling in BM. The RAs reprogrammed by melanoma cells expressed interleukin-23 to enhance the invasiveness of cancer cells, and cancer cell-derived matrix metalloproteinase-2 affected the status of astrocytes (48). In patient-derived xenografts of melanoma, polyunsaturated fatty acids, such as amino acid and mead acid, were found to be an RA-derived component to triggered proliferator-activated receptor signaling, hence boosting the growth of the tumor as well (49). Further analysis labeled a subpopulation of RAs with signal transducer and activator of transcription 3 (STAT3), providing the significant role of these RAs in BM. Additionally, microglia and macrophages could interact with RAs with activated STAT3 to create growth-promoting substances and prevent CD8^+^ T-cell activation (50).

The direct contact between RAs and brain-tropic cells and the pervasive communication through secreted substances play a part in the TIME of BM. Gap junction intercellular communication (GJIC) serves as a possible target and is crucial for preserving tissue homeostasis. Through the GJIC, calcium (Ca^2+^), an ion involved in homeostasis, is exchanged between RAs (51). Numerous gliotransmitters are said to be released as a result of the Ca^2+^ signaling. Due to the fact that it causes DNA damage, it is thought to be harmful to cancer cells (52). Lin et al. conducted co-culture experiments and found that the communication of Ca^2+^ between RAs and tumor cells process the ability to determine the fate of BM. The study suggested that direct physical contact rather than the factors secreted by RAs play a role in the chemoresistance of tumor cells. The proliferation and chemoresistance of melanoma to brain metastasis cells were aided by RAs’ capacity to remove Ca^2+^ from tumor cells (53). Additionally, recent studies proved that protocadherin 7 (PCDH7) expressed by breast and lung cancer cells could promote the carcinoma–astrocyte gap junction mediated by connexin 43 (Cx43). The gap junction, which sent the second messenger cGAMP from the cancer towards the astrocytes, may be substantially hampered by PCDH7 downregulation. Activating the STING pathway by cGAMP enables astrocytes to express inflammatory factors such as TNF-α and interferon-α. Consequently, STAT1 and NF-κB pathways are activated in metastatic cells to support their proliferation and chemoresistance (54). All the above evidence suggested that GJIC between RAs and malignant cells possess the ability to regulate and rebuild the TIME of BM.

As a result of a number of discoveries, it is clear that RAs play a crucial role in BM by interacting in a variety of ways and serving as a necessary part of the CNS. Malignant brain-tropic cells have taken control of RAs, making them no longer passive spectators. Research is still needed to fully understand the intricate structure of the BM microenvironment.

#### 2.1.3 Neuron is hijacked by tumor cells in BM

As highly specialized cells, the role of neurons in the CNS structure and function has been appreciated for a long time (55, 56). Neuron is the most important cell that processes high plasticity; it exerts functions such as action understanding, empathy, imitation, intention understanding, and language development in the CNS (57–59). Although extensive study has been done on the examination of neurons, it is still unclear how much function neurons have in BM.

Classical electrochemical communication is frequently mediated by neural chemicals released by neurons, such as neurotransmitters and neuropeptides (60). However, as revealed by recent studies, cancer-infected neurons operate as both a hub for information transmission and a driver of tumor growth and development (61, 62). The study by Deshpande et al. focused on the tumor–neuron interaction at the initial state of metastasis. By co-culturing neurons with breast and lung cancer cells, they tried to mimic the potential interaction between brain-seeking tumor cells and neurons and survey the induction of neurotransmitter and synaptic signaling. It was shown that neurons could cause the overexpression of neurotransmitters in tumors by observing the activation of genes for classical neurotransmitter receptors and neuronal synaptic mediators in tumors (63). The interaction between tumor and neurons allows one to target cancer in a neuron-dependent way. Cordero et al. engineered human neural stem cells LM008, which could continuously secrete functional antibodies against HER2 (anti-HER2Ab). By inducing similar effects to trastuzumab in HER2^+^ overexpressing breast cancer brain metastases (BCBM) models, the LM008 showed the ability to inhibit the proliferation of BCBM in the PI3K-Akt signaling pathway (64).

Additionally, a study of resected breast to brain metastasis (B2BM) specimens revealed a GABAergic phenotype comparable to neurons, which suggested that tumor cells escape their genetic restraints and are prepared for the microenvironment in the BM (65). As more is learned, the direct synapses between neurons and cancer cells aroused curiosity among scientists. According to Zeng et al., B2BM can construct pseudo-tripartite synapses with glutamatergic neurons, using the glutamate released by neurons to stimulate colony development *via* GluN2B-mediated N-methyl-D-aspartate receptors (NMDARs) (66). They demonstrated that NMDARs, a receptor involved in the transmission of nerve impulses, were upregulated on the cell membranes of B2BM as well (66). Additionally, as presented by Venkataramani et al., glutamatergic synapse also exerted its function in gliomas. Contrary to B2BM, malignant cell activity is promoted by the connections between presynaptic neurons and postsynaptic glioma cells *via* glutamate-mediated AMPA receptors (67). According to the studies mentioned above, BM may act like neurons to create connections in the CNS that will support the growth of that region. The findings show a bidirectional link between them and suggest that these signaling axes could one day lead to promising treatment options for BM.

#### 2.1.4 Tumor-infiltrating lymphocytes are the atypical cells in the BM

Lymphocytes, which develop from bone marrow stem cells, often serve as barriers against the invasion of various immune system disorders in the periphery (68). Regarding the CNS, the distinctive BBB structure restricts lymphocyte permeation and patrolling to, in the majority of cases, prevent additional damage to the CNS parenchyma (69).

However, with the impairment of the BBB’s integrity resulting from the malignant cell, lymphocytes could be recruited to the CNS and play bidirectional functions, which refers to restricting the metastasis of cancer cells or causing an inflammation in the brain parenchyma (70, 71). Fully understanding the complete scenario of CNS after the influx of lymphocytes caused by BM may yield an even more pronounced effect on developing new therapeutic regimens (72).

The presentation of TILs in the BM is sophisticated, in which the percentage and composition of tumor-infiltrating lymphocyte (TIL) subtypes vary a lot (73). Berghoff et al. conducted immunohistochemistry for biomarkers in the 116 BM specimens (61 from NSCLC BM). The findings demonstrated that the majority of samples exhibited infiltration of CD8^+^ TILs and dense CD3^+^ TILs, which suggested a potential successful TIME for immunotherapy; 26.2% of NSCLC specimens also had PD1^+^ TIL infiltration (74). TIL comparisons between BM and primary tumors revealed more data. In order to assess the expression of programmed death-ligand 1 (PD-L1), the profile of important T-cell subsets, activation and proliferation indicators, and coinhibitory receptors in lung and brain metastases, Lu et al. employed multiplexed quantitative immunofluorescence. In comparison to the main tumor, BM tissue displayed decreased levels of granzyme B, a marker for the efficiency of T cells, as well as CD3^+^, CD4^+^, CD8^+^, and FOXP3^+^ TILs. In addition, BM showed decreased amounts of T-cell immunoglobulin and mucin domain-containing protein 3, programmed death 1 (PD-1), and lymphocyte-activation gene 3 in CD3^+^ T cells (75).

#### 2.1.5 Others

**Figure 1:**
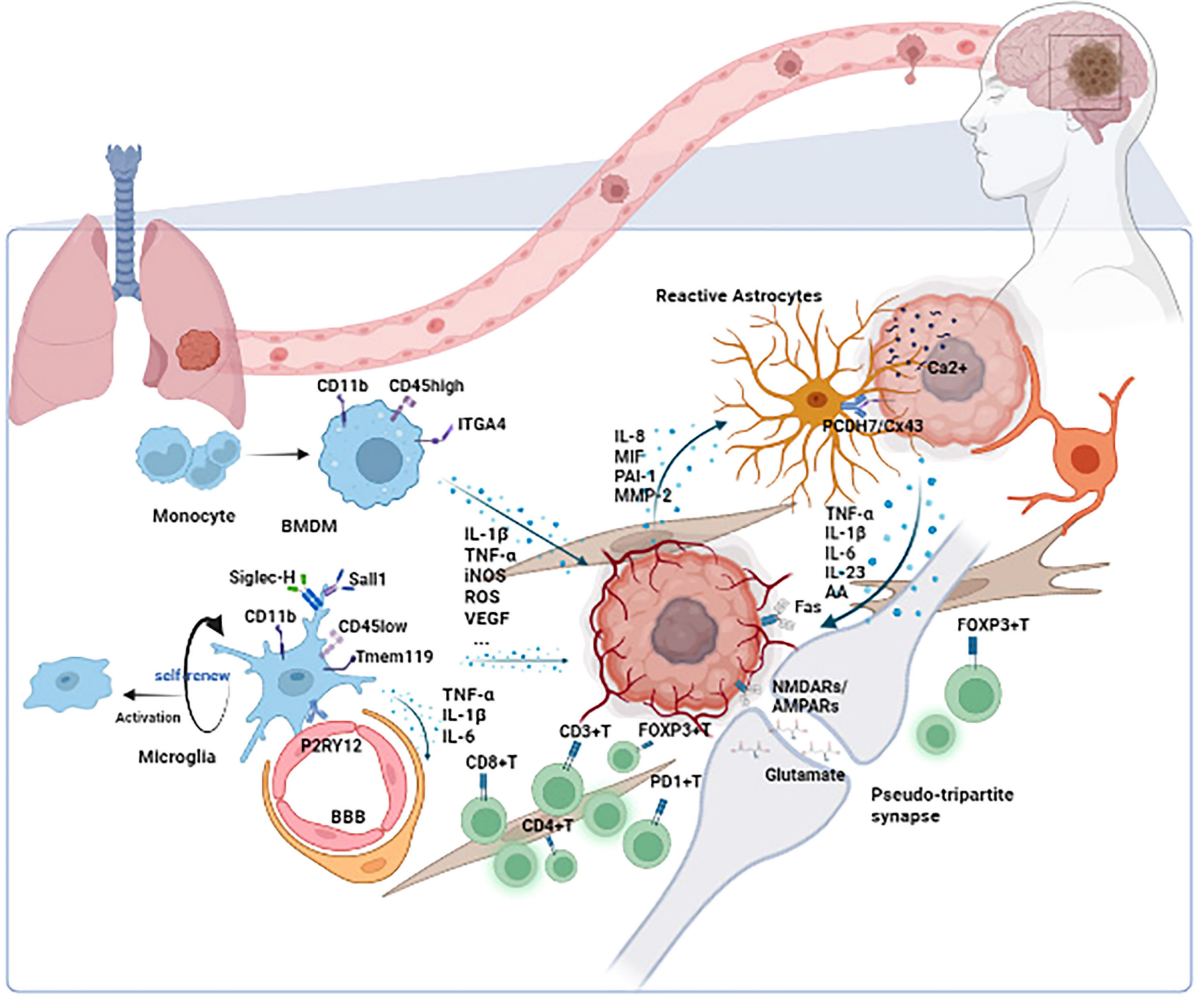
The scenario of TIME in BM. Microglia/BMDMs are the main macrophages in TIME of BM. Depending on the status of microglia/BMDMs, different cytokines such as IL-1β, TNF-α, and VEGF are secreted to influence the development of tumors. Astrocytes play their role through the paracrine cytokine signaling loops and direct contacts such as the PCDH7/Cx43 interaction. The tumor could also utilize the synapse between neuron and tumor to promote its development. Other cells such as lymphocytes, oligodendrocytes, fibroblasts, pericytes, and endothelial cells also function in TIME. TIME, tumor immune microenvironment; BM, brain metastasis; ITGA4, integrin subunit alpha 4; BMDM, bone marrow-derived macrophage; Siglec-H, sialic acid-binding immunoglobulin-like lectin H; Tmem 119, transmembrane protein 119; P2RY12, purinergic receptor P2Y, G-protein coupled, 12; BBB, blood–brain barrier; IL-1β, interleukin 1β; TNF-α, tumor necrosis factor α; iNOS, inducible nitric oxide synthase; ROS, reactive oxygen species; VEGF, vascular endothelial growth factor; IL-6, interleukin 6; IL-8, interleukin 8; MIF, migration inhibitory factor; PAI-1, plasminogen activator inhibitor-1; MMP-2, matrix metalloproteinase-2; PCDH7, protocadherin 7; Cx43, connexin 43; IL-23, interleukin 23; AA, amino acid; NMDARs, N-methyl-D-aspartate receptors; AMPARs, AMPA receptors.

Other BM cells presented in TIME of BM include oligodendrocyte, fibroblast, pericytes, and endothelial cells. All the above components play a unique role in BM’s development. Further analysis is needed. The scenario of TIME in BM is generalized in **Figure 1**.

### 2.2 Scenario of efficacy of ICIs in NSCLC with BM

#### 2.2.1 ICI monotherapy

KEYNOTE-024 was a phase III randomized clinical trial (RCT) that showed the significant superiority of pembrolizumab in advanced NSCLC with PD-L1 expression of at least 50% (7). The median OS (mOS) and median progression-free survival (mPFS) in pembrolizumab were 26.3 months and 7.7 months, compared with 13.4 months and 5.5 months in chemotherapy, respectively (76). However, in the subgroup analysis of brain metastasis patients, which includes 28 (9.2%) patients, the benefit of pembrolizumab was not significant (7). Moreover, Mansfield et al. conducted a pooled analysis of KEYNOTE-001, KEYNOTE-010, KEYNOTE-024, and KEYNOTE-042, which explored the outcomes of pembrolizumab in PD-L1-positive [tumor proportion score (TPS) ≥ 1%] NSCLC with BM (77). Among the 293 (9.2%) patients with BM, pembrolizumab showed a better OS and PFS in both TPS ≥ 1% [OS: hazard ratio (HR) 0.83 95% confidence interval (CI) 0.62–1.10; PFS: HR 0.96 95% CI 0.73–1.25) and TPS ≥ 50% (OS: HR 0.67 95% CI 0.44–1.02; PFS: HR 0.70 95% CI 0.47–1.03) subgroups, while the difference was not significant as well. Another analysis exploring the efficacy of pembrolizumab in patients with BM was conducted in a cohort of melanoma and NSCLC (11). This prospective study enrolled 37 (88.1%) NSCLC patients with PD-L1 expression of at least 1%, of whom the mPFS was 1.9 months (95% CI 1.8–3.7), and the mOS was 9.9 months (95% CI 7.5–29.8) (78).

Nivolumab, another anti-PD-1 antibody, yields similar results to pembrolizumab. In the subgroup analysis of CheckMate-078, 72 (14.3%) patients presented with BM. The nivolumab group containing 45 NSCLC experienced longer OS (HR 0.82 95% CI 0.62–1.10) (79). CheckMate-017, as well as CheckMate-057, explored the efficacy of nivolumab in patients with treated advanced NSCLC. With the success of both trials, nivolumab was approved by Food and Drug Administration and recommended by the National Comprehensive Cancer Network (80, 81). However, due to the limited number of patients with BM included in both trials, further analysis may conduct a large bias. Until recently, a pooled study of CheckMate-017, CheckMate-057, and CheckMate-063 that compared nivolumab with docetaxel showed that NSCLC with BM benefited more from nivolumab (mOS: 8.4 versus 6.2 months HR not mentioned) (82). The EMPOWER-Lung 1 trial examined the efficacy and safety of cemiplimab in untreated advanced NSCLC with PD-L1 at least 50% and enrolled 68 (12.1%) patients with treated BM. The analysis of mPFS (HR 0.45 95% CI 0.22–0.92) and mOS (HR 0.17 95% CI 0.04–0.76) showed that patients benefited significantly from cemiplimab, which showed more promising results to some degree (83).

The efficacy of atezolizumab in NSCLC with BM had been evaluated in OAK and FIR trials. OAK was a phase III RCT that explored the role of atezolizumab in previously treated advanced NSCLC and further demonstrated the potential benefit. A total of 85 patients were included in the subgroup analysis of BM, with 38 (8.9%) receiving atezolizumab, who yielded longer OS (20.1 *vs*. 11.9 months HR 0.54 95% CI 0.31–0.94) (84). FIR was a phase II trial evaluating atezolizumab, including 13 (9.4%) NSCLC presented with BM. The results showed that the objective response rate (ORR) was 23%, mPFS was 4.3 months, and mOS was 6.8 months in BM patients (85). The specific information of trials mentioned above and other retrospective studies (86–89) are presented in **Table 1**.

**Table 1:** Summary of ICI trials in NSCLC with BM.

Trial	Study type	Inclusion criteria	Arms	Overall patients		NSCLC with BM	
				No. of patients	Outcomes	No. of patients	Outcomes
ICI Monotherapy							
**KEYNOTE-024 (** 7, 76, 90) **(NCT02142738)**	Phase III RCT	Advanced NSCLC with PD-L1≥50%	Pembrolizumab versus Chemotherapy	154 versus 151	mPFS:7.7 m versus 5.5 m (HR 0.5, 95% CI 0.39–0.65); mOS:26.3 m versus 13.4 m (HR 0.62, 95% CI 0.48–0.81)	18 versus 10	mPFS: HR 0.55; 95% CI 0.20–1.56
**Mansfield et al. (** 77)	Pooled analysis	PD-L1 ≥ 1% advanced NSCLC	Pembrolizumab versus chemotherapy	3,170	NR	293	mOS:13.4 m versus 10.3 m (HR 0.83, 95% CI 0.62–1.10); mPFS:2.3 m versus 5.2 m (HR 0.96, 95% CI 0.73–1.25)
**Goldberg et al.** (78) **(NCT02085070)**	Phase II	Advanced NSCLC with BM	Pembrolizumab	NR	NR	42 (cohort 1: *n* = 37 PD-L1 ≥ 1%;cohort 2: *n* = 5 PD-L1 < 1%)	mPFS for cohort 1: 1.9 m (95% CI 1.8–3.7); mOS for cohort 1: 9.9 m (95% CI 7.5–29.8); 29.7% (95% CI 15.9–47.0) patients in cohort 1 had a BM response; 1-year OS was 40% (95% CI 30%–64%); 2-year OS was 34% (95% CI 21%–54%).
**CheckMate-078 (** 79) **(NCT02613507)**	Phase III RCT	Treated advanced NSCLC in the Chinese population	Nivolumab versus docetaxel	338 versus 166	mOS:12.0 m versus 9.6 m (HR 0.68, 97.7% CI 0.52–0.90); ORR: 17% versus 4%	45 versus 27	mOS: HR 0.82, 95% CI 0.62–1.10
**Goldman et al. (** 82)	Pooled analysis	Checkmate-017 (80); Checkmate-057 (81); Checkmate-067 (91)	Nivolumab versus docetaxel	427 versus 427^a^	mPFS: 2.5 m versus 3.5 m (HR 0.79, 95% CI 0.68–0.92); mOS: 11.1 m versus 8.1 m (HR 0.68, 95% CI 0.59–0.78); 5-year pooled OS: 13.4% (95% CI 10.4–16.9) versus 2.6% (95% CI 1.4–4.5); 5-year PFS: 8.0% (95% CI 5.4–11.2) versus 0% (92)	46 versus 42	mOS:8.4 m (95% CI 4.99–11.6) versus 6.2 m (95% CI 4.4–9.23)
**EMPOWER-Lung 1 (** 83)	Phase III RCT	Advanced NSCLC with PD-L1 ≥ 50%	Cemiplimab versus chemotherapy	283 versus 280	mPFS:8.2 m versus 5.7 m (HR 0.54, 95% CI 0.43–0.68); mOS: NR versus 14.2 m (HR 0.57, 95% CI 0.42–0.77)	34 versus 34	mPFS: HR 0.45, 95% CI 0.22–0.92; mOS: HR 0.17, 95% CI 0.04–0.76
**OAK (** 84) **(NCT02008227)**	Phase III RCT	Treated advanced NSCLC	Atezolizumab versus docetaxel	425 versus 425	mPFS: 2.8 m versus 4.0 m (HR 0.95, 95% CI 0.82–1.10); mOS:13.8 m versus 9.6 m (HR 0.73, 95% CI 0.62–0.87)	38 versus 47	mOS:20.1 m versus 11.9 m (HR 0.54, 95% CI 0.31–0.94)
**FIR (** 85) **(NCT01846416)**	Phase II	Advanced NSCLC	Atezolizumab	138 (cohort 1 = 31; cohort 2 = 93; cohort 3 = 13)	ORR: 32% versus 21% versus 23%; mPFS: 5.5 m versus 3.7 m versus 4.3 m; mOS: 14.4 m versus 9.3 m versus 6.8 m	13	ORR:23%; mPFS: 4.3 m; mOS: 6.8 m
**Dudnik et al. (** 86)	Retrospective study	Advanced NSCLC	Nivolumab	260	mOS: 5.9 m (95% CI 4.7–7.4)	55	mOS: 7.0 m (95% CI 4.7–10.8)
**EAP (** 87)	Retrospective study	Advanced treated squamous NSCLC	Nivolumab	371	mOS: 7.9 m (95% CI 6.2–9.6); mPFS: 4.2 m (95% CI 3.4–5.0); 1-year OS 39%; 1-year PFS 27%	37	mOS: 5.8 m (95% CI 1.8–9.8); mPFS: 4.9 m (95% CI 2.7–7.1); 1-year OS: 35%; 1-year PFS: 31%
**EAP (** 88)	Retrospective study	Advanced treated non-squamous NSCLC	Nivolumab	1,558	mOS: 11.3 m (95% CI 10.2–12.4); mPFS: 3.0 m (95% CI 2.9–3.1); 1-year OS: 48%; 1-year PFS: 22%	409	ORR: 17%; DCR: 39%; mOS: 8.6 m (95% CI 6.4–10.8); mPFS: 3.0 m (95% CI 2.3–3.3); 1-year OS: 43%; 1-year PFS: 20%
**Hendriks et al.** (89)	Retrospective study	Advanced ICI-treated NSCLC	PD1/PDL1 efficacy for BM versus non-BM	255 versus 770	ORR: 20.6% versus 22.7% (*p* = 0.484); mPFS: 1.7 m (95% CI 1.5–2.1) versus 2.1 m (95% CI 1.9–2.5) (*p* = 0.009); mOS: 8.6 m (95% CI 6.8–12.0) versus 11.4 m (95% CI 8.6–13.8) (*p* = 0.035)	255	ORR: 20.6%; mPFS: 1.7 m (95% CI 1.5–2.1); mOS: 8.6 m (95% CI 6.8–12.0)
ICI combined with chemotherapy							
**KEYNOTE-189 (** 8, 93, 94) **(NCT02578680)**	Phase III RCT	Advanced untreated non-squamous NSCLC	Pembrolizumab + chemotherapy versus placebo + chemotherapy	410 versus 106	mPFS: 9.0 m versus 4.9 m (HR 0.50, 95% CI 0.41–0.59); mOS: 22 m versus 10.6 m (HR 0.60, 95% CI 0.50–0.72)	73 versus 35	mPFS: 6.9 m versus 4.7 m (HR 0.42, 95% CI 0.27–0.67); mOS: 19.2 m versus 7.5 m (HR 0.41, 95% CI 0.24–0.67)
**Powell et al. (** 95)	Pooled analysis	Advanced untreated NSCLC	Pembrolizumab + chemotherapy versus placebo + chemotherapy	NR	NR	105 versus 66	mPFS: 6.9 m versus 4.1 m (HR 0.44, 95% CI 0.31–0.62); mOS: 18.8 m versus 7.6 m (HR 0.48, 95% CI 0.32–0.70)
**CameL (** 96) **(NCT03134872)**	Phase III RCT	Advanced untreated non-squamous NSCLC	Camrelizumab + chemotherapy versus chemotherapy	205 versus 207	mPFS: 11.3 m versus 8.3 m (HR 0.60, 95% CI 0.45–0.79); mOS: NR^e^ versus 20.9 m (HR 0.73, 95% CI 0.53–1.02)	11 versus 6	PFS: HR 0.14, 95% CI 0.01–0.88
**ORIENT-11 (** 97, 98) **(NCT03607539)**	Phase III RCT	Advanced untreated non-squamous NSCLC	Sintilimab + chemotherapy versus chemotherapy	266 versus 131	mPFS: 9.2 m versus 5.0 m (HR 0.49, 95% CI 0.38–0.63); mOS: NR versus 16.8 m (HR 0.60, 95% CI 0.45–0.79)	36 versus 22	PFS: HR 0.49, 95% CI 0.26–0.92; OS: HR 0.57, 95% CI 0.28–1.16
**Jiang et al.** (99) **(NCT03924050)**	Phase II	EGFR-TKI treated advanced NSCLC with no T790M mutation	Toripalimab + chemotherapy	40	mPFS: 7.0 m; mOS: 23.5 m; ORR: 50.0%; DCR: 87.5%	6	PR: 66.7%
**Atezo-Brain (** 100) **(NCT03526900)**	Phase II	Chemotherapy-naive stage IV non-squamous NSCLC with untreated BM	Atezolizumab + chemotherapy	NR	NR	40	12-week PFS rate: 67.1%; iPFS: 7.1 m (95% CI 4.6–11.2); mPFS: 8.9 m (95% CI 6.7–12.9)
ICI combined with radiotherapy							
**Wong et al. (** 101) **(NCT02978404)**	Phase II	NSCLC or RCC with BM	Nivolumab + SRS	26	NR	22	Median iPFS: 5.0 m; mPFS: 2.9 m; mOS: 14 m
**Singh et al. (** 102)	Retrospective study	NSCLC with BM receiving SRS	ICIs + SRS versus chemotherapy + SRS	NR	NR	39 versus 46	mOS: 10 m versus 11.6 m (*p* = 0.23)
**Minniti et al. (** 103)	Retrospective study	NSCL and melanoma BM receiving postoperative SRS	SRS + ICIs versus SRS	63 versus 66	mOS: 24.8 m versus 14.7 m (*p* = 0.007); 1-year OS rate 78% versus 58.7%; 2-year OS rate: 50% versus 22.8%; 1-year extracranial PFS rates: 59% versus 43%; 2-year extracranial PFS rates: 39% versus 19%	27 versus 29	2-year OS and DBF^b^: 44% and 29%
**Scoccianti et al. (** 104)	Retrospective study	NSCLC with BM receiving SRT	SRT + ICIs versus SRT	NR	NR	100 versus 50	SRT + ICIs: iLPFS^c^ was 89.5% and 83.9%; iDPFS^d^ was 69.7% and 55.2%; OS was 79.4% and 64.5% at 6 and 12 months
**Enright et al. (** 105)	Retrospective study	NSCLC with BM receiving SRT	SRT + ICIs versus SRT	NR	NR	33 versus 44	2-year local control: 97% versus 86% (*p* = 0.046); 2-year DBF was 38.6% versus 66.5% (*p* = 0.016); 1-year OS rate: 68% versus 64%; 2-year OS rate: 62% versus 35%
Others							
**CheckMate-227 (** 106, 107) **(NCT02477826)**	Phase III RCT	Advanced untreated NSCLC	Nivolumab + ipilimumab versus chemotherapy	583 versus 583	mOS: 17.1 m (95% CI 15.2–19.9) versus 13.9 m (95% CI 12.2–15.1)	69 versus 66	mPFS: 5.4 m versus 5.8 m (HR 0.79, 95% CI 0.52–1.19); mOS: 18.8 m versus 13.7 m (HR 0.57, 95% CI 0.38–0.85)
**CheckMate-9LA (** 108, 109) **(NCT03215706)**	Phase III RCT	Advanced untreated NSCLC	Nivolumab + ipilimumab + chemotherapy versus chemotherapy	361 versus 358	mOS: 15.6 m versus 10.9 m (HR 0.66, 95% CI 0.55–0.80)	51 versus 50	mPFS: 10.6 m versus 4.1 m (HR 0.40, 95% CI 0.25–0.64); iPFS: 13.5 m versus 4.6 m (HR 0.36, 95% CI 0.22–0.60); mOS: 19.3 m versus 6.8 m (HR 0.43, 95% CI 0.27–0.67)
**Chu et al. (** 110) **(NCT03628521)**	Phase Ib	Advanced untreated NSCLC	Sintilimab + anlotinib	22	ORR: 72.7% (95% CI 49.8%–89.3%); DCR: 100% (95% CI 84.6%–100%); mPFS: 15 m (95% CI 8.3–NR)	4	PR: 3/4; SD: 1/4

a The patients included in the outcome analysis of overall patients only pooled Checkmate-017 and Checkmate-057.

b Distant brain failure (DBF).

c Intracranial local progression-free survival (iLPFS).

d Intracranial distant progression-free survival (iDPFS).

All the trials above suggest that although advanced NSCLC benefits significantly from ICI monotherapy, the improvement of survival benefit to BM patients from ICI monotherapy is still finite, presented with the mPFS varying from 1.7 months to 4.9 months, and mOS varying from 20.1 months to 5.8 months. Further RCTs are needed to explore the efficacy of ICI monotherapy in NSCLC with BM.

#### 2.2.2 ICI combined with chemotherapy

Due to the slow onset of the anti-tumor immune response and the limited efficacy of ICI monotherapy in NSCLC patients with BM, the synergy effects between ICIs and chemotherapy aroused the interest of scientists. Many trials revealed that chemotherapy helps improve the efficacy of immunotherapy for NSCLC patients with BMs.

KEYNOTE-189 revealed that advanced non-squamous NSCLC benefits from adding pembrolizumab to chemotherapy in first-line treatment, with an mPFS and mOS of 9.0 months and 22 months, respectively (93). As for the BM subgroup, 73 (67.6%) patients were in the ICI-based combination group, while 35 (32.4%) were in the chemotherapy group, and significant prolonged PFS (6.9 vs. 4.7 months HR 0.42 95% CI 0.27–0.67) and OS (19.2 *vs*. 7.5 months HR 0.41 95% CI 0.24–0.67) were observed, which suggested the promising future for the combination of ICIs and chemotherapy (111). The subgroup analysis of BM patients in KEYNOTE-407 was not reported, which explored similar treatment regimes in advanced squamous NSCLC (112). Because of the limited data on the efficacy of pembrolizumab–chemotherapy for NSCLC with BM, Powell et al. conducted a pooled analysis based on KEYNOTE-021, KEYNOTE-189, and KEYNOTE-407. A total of 171 NSCLC patients with BM were enrolled in this study, and the results were similar to KEYNOTE-189, which showed that platinum-doublet chemotherapy with the addition of pembrolizumab not only prolonged the PFS (6.9 vs. 4.1 months, HR 0.44, 95% CI 0.31–0.62) but also improved the OS (18.8 vs. 7.6 months, HR 0.48, 95% CI 0.32–0.70) (95).

Several domestic regimes such as camrelizumab, sintilimab, toripalimab, and tislelizumab achieve promising results nowadays. Seventeen patients with BM were enrolled in CameL, a trial comparing the efficacy of camrelizumab plus chemotherapy with chemotherapy alone in previously untreated non-squamous NSCLC, and the results showed that the ICI combination group benefits more in PFS (HR 0.14, 95% CI 0.01–0.88) (96). ORIENT-11 obtained similar results as well; advanced non-squamous NSCLC patients with BM (58/397) benefited from sintilimab plus pemetrexed-platinum more with a prolonged PFS (HR 0.49, 95% CI 0.26–0.92) and OS (HR 0.57, 95% CI 0.28–1.16), though the difference of OS was not significant (97). However, for the squamous NSCLC, the subgroup analysis of BM patients was not reported in both clinical trials (CameL-sq and ORIENT-12) (113, 114). As for toripalimab and tislelizumab, results for the untreated NSCLC with BM were not reported yet. A phase II study exploring the toripalimab in EGFR-TKI treated patients is presented in **Table 1** (99).

Atezo-Brain was a single-arm phase II study reporting the efficacy of atezolizumab, an anti-PD-L1 antibody, in untreated non-squamous NSCLC patients with BM. A total of 40 patients were included, and the systemic PFS and intracranial PFS (iPFS) were 8.9 months (95% CI 6.7–12.9) and 7.1 months (95% CI 4.6–11.2), respectively (100). The detailed information of trials is presented in **Table 1**.

#### 2.2.3 ICI combined with radiotherapy

As the standard localized therapy for BM patients, radiotherapy could relieve nervous symptoms quickly; thus, the combination of radiotherapy and ICI possibly form the best alliance in clinical practice. A phase II trial enrolling 22 (84.6%) NSCLC with BM evaluated the efficacy and safety of the combination of nivolumab and stereotactic brain radiosurgery. The results showed that upfront SRS during nivolumab in NSCLC was tolerated, and the median iPFS and systematic PFS were 5 months and 2.9 months, respectively (101). Singh et al. conducted a retrospective study assessing the local tumor response and survival outcomes in advanced NSCLC between the group of ICIs plus SRS versus chemotherapy plus SRS. Although the OS and the safety between the two groups showed no significant difference (10 *vs*. 11.6 months, *p* = 0.23), ICIs plus SRS presented with a more significant lesion shrank (90% vs. 47.8%, *p* = 0.001) among the subgroup of patients with brain lesions larger than 500 mm^3^ (102). Minniti et al. conducted a similar study on NSCLC and melanoma patients receiving postoperative SRS with or without ICIs. The addition of ICIs to SRS significantly prolonged the OS and the PFS. Except for the outcomes above, this study focused on controlling leptomeningeal disease (LMD) and found that the combination decreased the incidence of LMD (103). Compared with SRS, stereotactic radiotherapy (SRT) showed its advantage in reducing the occurrence of radiation necrosis. Two similar retrospective analyses compared SRT plus ICIs versus SRT alone, and the results showed that the combination group improved the OS and local control with a safe profile (104, 105). The detailed information of trials is presented in **Table 1**.

#### 2.2.4 Others

Except for the therapies mentioned above, others, such as multiple ICIs combination and anti-angiogenic agent combination, have also been thought to produce synergistic effects to NSCLC patients with BM. A *post-hoc* analysis focusing on the efficacy of ICI therapy in patients with BM of CheckMate-227 revealed that double-ICI regimens, nivolumab plus ipilimumab, worked well, resulting in prolonged OS (18.8 vs. 13.7 months, HR 0.57, 95% CI 0.38-0.85) compared with chemotherapy (106). Another trial exploring the dual-ICI therapy was CheckMate-9LA. Different from CheckMate-227, CheckMate-9LA compared the efficacy of nivolumab plus ipilimumab and two-cycle chemotherapy with four-cycle chemotherapy in advanced NSCLC (108). The results showed that ICIs–chemotherapy has an advantage over chemotherapy alone in the BM subgroup, presenting with a longer OS (19.3 *vs*. 6.8 months, HR 0.43, 95% CI 0.27–0.67) and PFS (10.6 vs. 4.1 months, HR 0.40, 95% CI 0.25–0.64) (109). As for the combination of anti-angiogenic regimes and ICIs, Chu et al. conducted a phase Ib study exploring the efficacy and safety of sintilimab plus anlotinib in advanced NSCLC. The trial enrolled 4 (18.2%) patients with BM, and 3/4 BM patients achieved partial response (PR) (110). The promising results above suggested the underlying value of multimodality ICI-based therapy in NSCLC with BM. However, it should be noted that there is a long way to go in this area. The detailed information of these trials is presented in **Table 1**.

## 3 Discussion and prospects

Patients with BM from lung cancer have poor prognoses and few therapy choices. Immunotherapy has entered a new era, and ICIs have ushered in a new era of treatment for this subgroup. We will be better able to comprehend the mechanism underlying the effectiveness of immunotherapy when we fully mine the TIME features of BM. The primary elements of the brain immune microenvironment are microglia and BMDMS, which have both direct and indirect impacts on the growth of brain metastases through a variety of cytokines. Microglia/BMDMS perform cytotoxic action, phagocytosis, and antigen presentation as part of their immunological function. Future research on this topic will concentrate on understanding how to switch microglia/BMDMS from the tumor-promoting M2 phase to the tumor-suppressing M1 phase. Changes in the functional status of astrocytes, which are the stromal cells of the central nervous system, have varying impacts on the microenvironment of brain metastases. According to studies, reactive astrocytes help create a fibrotic tumor brain microenvironment that is unfavorable to the effects of immunotherapy (115). Additionally, one of the significant elements influencing the effectiveness of immunotherapy is the quantity and activity of lymphocytes in brain metastases, and efficient T cells contribute to this improvement (116).

We also outlined the ongoing clinical trials on NSCLC with BM from the viewpoints of a single medicine, immune combined radiation, immune combined chemotherapy, and dual immune combination based on the existing clinical research. The efficacy and safety of ICIs in patients with BM are similar to those in the general population, according to an increasing body of study data. However, it is also important to keep in mind that most studies only assess patients with BM as a small subgroup; further research in this area is still required.

## Author contributions

MX: Manuscript writing. CS: Conceptualization and manuscript revision. All authors contributed to the article and approved the submitted version.
